# Prevalence and Distribution of High-Risk Genotypes of HPV in Women with Severe Cervical Lesions in Madrid, Spain: Importance of Detecting Genotype 16 and Other High-Risk Genotypes

**DOI:** 10.4061/2011/269468

**Published:** 2010-09-27

**Authors:** Maria Luisa Mateos Lindemann, Juan Manuel Sánchez Calvo, Jesús Chacón de Antonio, Itziar Sanz, Esperanza Diaz, Maria Dolores Rubio, Maria Luisa de la Morena

**Affiliations:** ^1^Department of Microbiology, Ramón y Cajal Hospital, Carretera de Colmemar Viejo Km 9.1, 28034 Madrid, Spain; ^2^Department of Pathology, Ramón y Cajal Hospital, Carretera de Colmemar Viejo Km 9.1, 28034 Madrid, Spain; ^3^Department of Gynaecology, Ramón y Cajal Hospital, Carretera de Colmemar Viejo Km 9.1, 28034 Madrid, Spain

## Abstract

*Background*. Persistent infection with high-risk human papillomavirus (HR-HPV) has been demonstrated to be the necessary causal factor for developing cervical cancer. To know the most prevalent HR-HPV in different geographical areas is important to design diagnostic tests and implementation of vaccines. *Objectives*. The goal of this study is to evaluate the prevalence of HR-HPV in a total of 1001 patients, 198 with normal cytology results, 498 with low-grade squamous intraepithelial lesion (LSIL), and 205 with high-grade squamous intraepithelial lesion (HSIL) who attended our gynaecology department for opportunistic screening of HPV infection. *Study design*. Cervical samples were taken in a PreservCyt vial (Cytyc Corporation, Boxborough, MA). Hybrid capture assay was carried out following the manufacturer's instructions (Digene Corp., Gaithersburg, MD). All samples were further studied with polymerase chain reaction (PCR) (Linear Array HPV Genotyping Test, Roche Diagnostics, Mannheim, Germany). *Results*. Genotype 16 was the most prevalent HR-HPV in the three groups, 17.8% in the patients with normal cytology results, 22.3% in the LSIL group, and 60% in the HSIL group. Genotype 18 had a very low prevalence in all groups. Other HR-HPV genotypes such as genotype 31, genotype 58 and genotype 52 were found in significant numbers in HSIL patients. *Discussion*. Our data show that genotypes 16, 31, 58, and 52 are the most prevalent HR-HPV in cervical samples with severe intraepithelial lesion in Spain. There may be some geographical variation in prevalence of carcinogenic types, and it must be considered for designing diagnostic tests and vaccine.

## 1. Background

Persistent infection with some genotypes of human papillomavirus (HPV) is the cause of cervical cancer. This virus comprises more than 100 genotypes of which 12 have been recognized as clearly oncogenic viruses and defined as “high risk-genotypes” (HR-HPV) (16, 18, 31, 33, 35, 39, 45, 51, 52, 56, 58, and 59). In addition, six more genotypes have been defined as “probably high risk” (26, 53, 66, 68, 73, and 82) [1]. There is increasing evidence that some HR-HPVs are more carcinogenic than others, particularly genotypes 16, 18, and 45 [2], and therefore, some authors have suggested that women older than 30 years with negative cytological results and infected with genotype 16 or 18 must be referred for colposcopy [3]. Consequently, the specific HR-HPV genotype detected is an important indicator of the risk for developing high-grade squamous intraepithelial lesion or greater (HSIL).

For these reasons and considering that HPV immunity is genotype specific, it is important to define HR-HPV prevalence in different geographical regions. To our knowledge there are limited data concerning the distribution of HR-HPV in Spanish women with precancerous or >HSIL lesions.

## 2. Objectives

This study aimed to define HR-HPV genotypes present in women with histological confirmed HSIL in comparison with HR-HPV genotypes present in women with mild cervical lesions or low-grade squamous intra-epithelial lesion (LSIL). Another goal of our study was to investigate whether genotypes 16 and 18 are the most prevalent in women with histological abnormalities in order to ascertain the benefits of vaccine implementation in our country and choice of diagnostic tests.

## 3. Study Design

### 3.1. Patients

From April 2006 to April 2009, 1001 consecutive women attending the Gynaecologic Department of Ramón y Cajal Hospital (Madrid, Spain) were prospectively included in this study. All the patients underwent a cervical swab for cytology and HPV detection taken on the initial visit to the gynaecology consultation. Only women with positive hybrid capture assay (HC2) results and available cytology results confirmed with histology were included in the study. If both results were discordant, histological results were considered. Results were recorded following the 2001 Bethesda System terminology [4]^.^

### 3.2. Methods

Cervical samples were taken and washed in a Preservcyt vial (Cytyc Corporation, Boxborough, MA) and sent to the Virology Unit of Ramón y Cajal Hospital for HPV detection. HC2 assay was carried out following the manufacturer's instructions after using a sample conversion kit (Digene Corp., Gaithersburg, MD). The samples were examined for the presence of HR-HPV types 16, 18, 31, 33, 35, 39, 45, 51, 52, 56, 58, 59, and 68. Positive and negative controls were included in each run.

All positive samples were further studied with polymerase chain reaction (PCR) (Linear Array HPV Genotyping Test, Roche Diagnostics, Mannheim,Germany). DNA extraction was performed automatically with a method validated previously in [5]. The primer pair PGMY09/11 included in this test is designed to detect 37 genotypes including 13 HR-HPVs (6, 11, 16, 18, 26, 31, 33, 35, 39, 40, 42, 45, 51, 52, 53, 54, 55, 56, 58, 59, 61, 62, 64, 66, 67, 68, 69, 70, 71, 72, 73, 81, 82, 83, 84, CP6108, and IS39). The human beta-globin gene is amplified as internal control. After the hybridization reaction, the strips were read visually with a reference guide.

### 3.3. Statistical Methods

Summary statistics (*n*, mean, standard error, median, min, max) for age at study entry by cytological and histological results were produced (Table 1). Patients with cervical intraepithelial neoplasia grade 3 (CIN3) results were later analyzed separately for a more accurate interpretation of the data. Mean age among cytological and histological results was compared using the Kruskal-Wallis test. Prevalence of HR-HPV types by cytological and histological results (normal, LSIL, HSIL, and CIN3) was computed irrespective of the presence of mixed infections (more than one HR-HPV genotype) (Table 2).

## 4. Results

We found 298 women with normal cytology, 498 with LSIL, and 205 with HSIL. CIN 3 was recorded in 111/205 patients from the HSIL group (Table 2), and these samples were studied separately since they represent the real target of cervical cancer screening campaigns.

As shown in Table 2, the most prevalent HR-HPV types in patients with normal cytology were HPV-16 (17.8%) followed by HPV-31 (12.8%) and HPV-52 (12.4%). In total, 17 HR-HPV types were represented. In the LSIL group of patients, the most prevalent HR-HPV types were HPV-16 (22.3%), HPV-53 (15.9%), and HPV-51 (14.1%). In women with HSIL cytological results the most prevalent HR-HPV types found were HPV-16 (60%), HPV-31 (15.1%), and HPV-58 (12.2%). If only patients with CIN3 lesions were selected, the predominant genotypes were the same with slight differences, HPV-16 (64%) followed by HPV-31 (14%), and HPV-58 (11.4%). Genotypes 16, 31 and 58 were more prevalent in CIN3 (64%, 14%, and 11.4%. resp.) than in LSIL patients ( 22.3%, 12.7%, and 8.2%, resp.) although the difference was only statistically significant for genotype 16 (CIN3 : LSIL prevalence ratio: 8.43, 95% CI: 5.41–13.13 for genotype 16, CIN3 : LSIL prevalence ratio 1.16, 95% CI: 0.64–2.10 for genotype 31 and CIN3:LSIL prevalence ratio 1.48 95% CI: 0.76–2.86 for genotype 58). Genotypes 16 or 18 were not present in 33 patients (29%) and the most frequent HR-HPV genotypes in these patients were 31, 58, 33 and 52 (10%, 10%, 7% and 5%, resp.). Only one LR-HPV, genotype 84, was found as a single type in only one CIN3 patient. The average age of women with LSIL and HSIL was 35.3 and 39.1 years, respectively (Table 1). This difference is statistically significant (*P* < .0001) age.

## 5. Discussion

Our cross-sectional study was performed on patients referred to the gynaecology office seeking for an opportunistic cervical cancer screening. Although these results cannot be extrapolated to a population-based screening, some interesting data were found. To establish the prevalence and distribution of HR-HPV in different geographical areas is very important in order to predict the benefits of vaccination and to design cancer screening programs with diagnostic tests including the most prevalent genotypes in each geographical area. Smith et al. [6] have performed a meta-analysis study and reported that genotypes 16, 18, 31, 33, 35, 45, 52, and 58 are the most prevalent types detected in a total of 14 595 invasive cervical cancer and 7 094 HSIL cases. However their relative importance differed in several countries in women with CIN3 lesions. In general, HR-HPV 16 and 18 are considered the two most prevalent genotypes in patients with CIN3 lesions or more severe. On the other hand recent reports have shown worldwide geographical variation in the prevalence patterns of HR-HPV specially genotype 18. For instance, in Italy, the predominant HR-HPV types are genotypes 16 (71%), 31 (10%), and 51 (9%). Genotype 18 was only found in 6% of the patients [7]. In contrast, in Australia, 18.3% of biopsies from women with severe lesions contained genotype 18 [8]. In our study, using the same primers for the PCR assay (PGMY09/11) as in this Australian study, genotype 18 was only found in 7 (6.1%) of the patients with >CIN3. This genotype is also uncommon in China [9]. Interestingly, genotype 31, the second most prevalent in our study, is also very frequent in Italy [7] and in the United States [10] but not in other countries [8].

The failure to detect exclusively low-risk genotypes in high-grade dysplasias has been reported by other authors [8]. We found only one patient with CIN3 associated to the LR-HPV genotype 84.

As expected the mean age of patients with HSIL is higher than the mean age of patients with LSIL.

In conclusion, geographical variation in prevalence of HR-HPV exits and is an important factor to consider in the implementation of vaccine campaigns and design of new diagnostic assays. Our study describes the HR-HPV distribution in 1001 infected women with available cytological and histological results in Madrid, Spain. Regarding the group of patients who developed CIN3 lesions, our data show that genotype 16 is the most prevalent in our country but other HR-HPVs such as genotypes 31, 58, 33, and 52 must also be targeted in the design of diagnostic tests and prophylactic vaccines. More information about prevalence of HR-HPV in different areas of the world is needed to predict how HPV vaccine and the screening with an HPV test will influence cervical cancer prevention.

## Figures and Tables

**Table 1:** Summary statistics for age by histological results.

Histology^a^	*N* (%)	Mean ± SE	Median	Min	Max
Normal	298 (29.8%)	36.5 ± 0.7	34	16	79
LSIL^b^	498 (49.7%)	35.3 ± 0.5	33	16	77
HSIL^c^	205 (20.5%)	39.1 ± 0.8	37	18	82

^a^Kruskal-Wallis test, *P* < .0001.

^b^Low squamous intraepithelial lesion.

^c^High squamous intraepithelial lesion.

**Table 2:** Distribution of HR-HPV types by histological and cytological diagnosis.

HPV type	NORMAL (*n* = 298) *N* (%)	LSIL^a^ (*n* = 498) *N* (%)	HSIL^b^ (*n* = 205) *N* (%)	CIN3^c^ (*n* = 111) *N* (%)	Total cases (*n* = 1001)
HPV-16	53 (17.8%)	111 (22.3%)	123 (60%)	73 (64%)	287 (28.7%)
HPV-18	21 (7%)	26 (5.2%)	11 (2.4%)	7 (6.1%)	58 (5.8%)
HPV-26	0 (0%)	8 (1.6%)	0 (0%)	0 (0%)	8 (0.8%)
HPV-31	38 (12.8%)	63 (12.7%)	31 (15.1%)	16 (14%)	132 (13.2%)
HPV-33	18 (6%)	41 (8.2%)	16 (7.8%)	7 (6.1%)	75 (7.5%)
HPV-35	10 (3.4%)	21 (4.2%)	6 (2.9%)	4 (3.5%)	37 (3.7%)
HPV-39	18 (6%)	39 (7.8%)	7 (3.4%)	2 (1.8%)	64 (6.4%)
HPV-45	11 (3.7%)	26 (5.2%)	6 (2.9%)	3 (2.6%)	43 (4.3%)
HPV-51	29 (9.7%)	72 (14.5%)	9 (4.4%)	3 (2.6%)	110 (11%)
HPV-52	37 (12.4%)	52 (10.4%)	19 (9.3%)	9 (7.9%)	108 (10.8%)
HPV-53	30 (10.1%)	79 (15.9%)	11 (5.4%)	3 (2.6%)	120 (12%)
HPV-56	23 (7.7%)	56 (11.2%)	10 (4.9%)	4 (3.5%)	89 (8.9%)
HPV-58	19 (6.4%)	41 (8.2%)	25 (12.2%)	13 (11.4%)	85 (8.5%)
HPV-59	14 (4.7%)	41 (8.2%)	7 (3.4%)	3 (2.6%)	62 (6.2%)
HPV-66	23 (7.7%)	61 (12.2%)	5 (2.4%)	2 (1.8%)	89 (8.9%)
HPV-68	7 (2.3%)	19 (3.8%)	3 (1.5%)	0 (0%)	29 (2.9%)
HPV-73	12 (4%)	29 (5.8%)	5 (2.4%)	1 (0.9%)	46 (4.6%)
HPV-82	3 (1%)	6 (1.2%)	3 (1.5%)	1 (0.9%)	12 (1.2%)

^a^Low squamous intraepithelial lesion in cytology.

^b^High squamous intraepithelial lesion in cytology.

^c^Cervical intraepithelial neoplasia of grade 3 or worse in histology.
